# 1-Benzyl-3-methyl­imidazolium chloride 0.25-hydrate

**DOI:** 10.1107/S1600536809053306

**Published:** 2009-12-19

**Authors:** Xiujie Ji, Bowen Cheng, Jun Song, Chao Liu

**Affiliations:** a Tianjin Municipal Key Laboratory of Fiber Modification and Functional Fibers, Tianjin Polytechnic University, Tianjin 300160, People’s Republic of China; b School of Materials Science and Engineering, Hebei University of Technology, Tianjin 300130, People’s Republic of China

## Abstract

The asymmetric unit of the title compound, C_11_H_13_N_2_^+^·Cl^−^, contains two independent ion pairs and and half a solvent water mol­ecule (*m* site symmetry for the O atom). The imidazole ring is oriented at dihedral angles of 66.61 (3) and 89.17 (3)° with respect to the aromatic ring in the two cations. In the crystal, O—H⋯(O,Cl) hydrogen bonds and π–π stacking inter­actions between the imidazole ring of one mol­ecule and the aromatic ring of another [perpendicular distance = 3.4 (4) Å] link the mol­ecules.

## Related literature

For general background to ionic liquids, see: Fukaya *et al.* (2006 ▶); Ranu *et al.* (2005 ▶); Chen *et al.* (2005 ▶); Blanchard *et al.* (2001 ▶); Zhang *et al.* (2006 ▶); Katayanagi *et al.* (2004 ▶). For bond-length data, see: Allen *et al.* (1987 ▶).
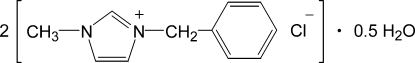


## Experimental

### 

#### Crystal data

C_11_H_13_N_2_^+^·Cl^−^·0.25H_2_O
*M* *_r_* = 213.19
Orthorhombic, 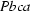

*a* = 11.452 (2) Å
*b* = 11.410 (2) Å
*c* = 34.867 (7) Å
*V* = 4555.8 (16) Å^3^
*Z* = 16
Mo *K*α radiation
μ = 0.30 mm^−1^
*T* = 113 K
0.22 × 0.20 × 0.16 mm

#### Data collection

Rigaku Saturn diffractometer
Absorption correction: multi-scan (*CrystalClear*; Rigaku, 2000 ▶) *T*_min_ = 0.937, *T*_max_ = 0.953
30046 measured reflections
4022 independent reflections
3619 reflections with *I* > 2σ(*I*)
*R*_int_ = 0.041

#### Refinement

*R*[*F* ^2^ > 2σ(*F* ^2^)] = 0.042
*wR*(*F* ^2^) = 0.119
*S* = 1.08
4022 reflections
268 parameters
3 restraints
H atoms treated by a mixture of independent and constrained refinement
Δρ_max_ = 0.30 e Å^−3^
Δρ_min_ = −0.28 e Å^−3^

### 

Data collection: *CrystalClear* (Rigaku, 2000 ▶); cell refinement: *CrystalClear*; data reduction: *CrystalClear*; program(s) used to solve structure: *SHELXS97* (Sheldrick, 2008 ▶); program(s) used to refine structure: *SHELXL97* (Sheldrick, 2008 ▶); molecular graphics: *SHELXTL* (Sheldrick, 2008 ▶) and *PLATON* (Spek, 2009 ▶); software used to prepare material for publication: *SHELXTL*.

## Supplementary Material

Crystal structure: contains datablocks global, I. DOI: 10.1107/S1600536809053306/bq2176sup1.cif

Structure factors: contains datablocks I. DOI: 10.1107/S1600536809053306/bq2176Isup2.hkl

Additional supplementary materials:  crystallographic information; 3D view; checkCIF report

## Figures and Tables

**Table 1:** Hydrogen-bond geometry (Å, °)

*D*—H⋯*A*	*D*—H	H⋯*A*	*D*⋯*A*	*D*—H⋯*A*
O1—H1*E*⋯O1^i^	0.86 (1)	1.81 (4)	2.434 (7)	128 (4)
O1—H1*E*⋯Cl2^i^	0.86 (1)	2.51 (3)	3.179 (3)	135 (4)

Symmetry code: (i) 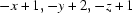.

**Fig. 1.**
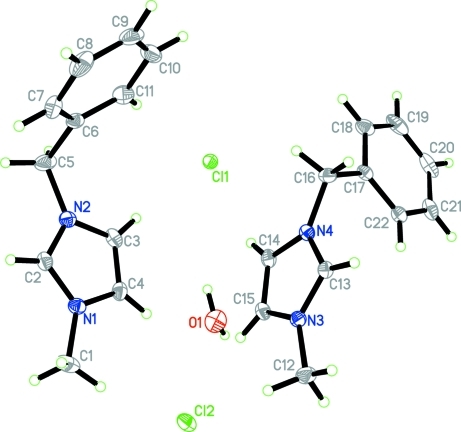
The molecular structure of the title molecule, with the atom-numbering scheme. Displacement ellipsoids are drawn at the 30% probability level.

**Fig. 2.**
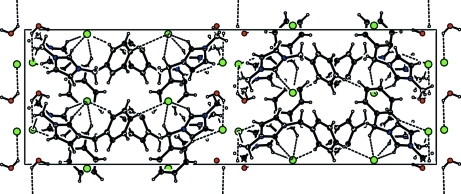
The packing diagram of (I).

## supplementary crystallographic information

### Comment

Ionic liquids (ILs) are a class of compounds composed of organic cations and organic or inorganic anions and have attracted significant attention due to their beneficial properties and their low impact on environment. Many investigations have been dedicated to their use as new media for synthetic chemistry (Fukaya *et al.*, 2006), catalysts for organic synthesis (Ranu *et al.*, 2005; Chen *et al.*, 2005), extractants for separation science (Blanchard *et al.*, 2001) and electrolytes for electrochemistry (Zhang *et al.*, 2006) as well as other areas due to their peculiar physical properties such as a wide liquid range, non-volatility, chemical stability and large windows of electrochemistry. Because it is technically difficult for ionic liquid to grow single crystal and to select suitable sample for single crystal x-ray diffraction at low temperature, so far a few crystal structures have been determined by x-ray diffraction (Katayanagi *et al.*, 2004). We have synthesized a numer of ionic liquids. The title compound is one of the products, and we report herein its crystal structure.

In the molecule of the title compound (Fig. 1) the bond lengths (Allen *et al.*,  1987) and angles are within normal ranges. The imidazole ring is oriented with respect to the aromatic ring at a dihedral angle of 89.17 (3)° and 66.61 (3)°, respectively. The hydrogen bond are formed between one Cl and H_2_O (Table 1). In the crystal structure, π-π packing between the imidazole ring of one molecule and the aromatic ring of the other [perpendicular distance = 3.4 (4)Å] link the molecules. The packing diagram of the O-H···Cl bonds is shown in Fig. 2.

### Experimental

For the preparation of the title compound, N-methylimidazole (8.2 g, 0.1 mol) was dissolved in dry acetonitrile (30 ml). Benzyl chloride (12.7 g, 0.1 mol) was added to this solution, the reaction was stirred under reflux for 7 h. The reaction mixture was extracted with ethyl acetate. After concentration, the residue was purified by recrystallization from chloroform (yield; 19.4 g, 93%, m.p. 251 K). Spectroscopic analysis: IR (KBr, ν, cm^-1^): 3415, 3085, 2927, 1634, 1571, 1455, 1160, 824, 722. Analysis required for C_22_H_27_Cl_2_N_4_O_0.5_: C 61.97; H 6.38; N 13.14%. Found: C 61.92; H 6.40; N 13.10%.

### Refinement

H atoms of water were located in a different Fourier map and the atomic coordinates allowed to refine freely. Other H atoms were positioned geometrically and refined as riding (C-H = 0.93-0.96Å) and allowed to ride on their parent atoms, with U_iso_(H) =1.2U_eq_(parent) or 1.5U_eq_(parent).

### Crystal data

**Table:**

C_11_H_13_N_2_^+^·Cl^−^·0.25(H_2_O)	*D*_x_ = 1.243 Mg m^−^^3^
*M**_r_* = 213.19	Melting point: 251 K
Orthorhombic, *P**b**c**a*	Mo *K*α radiation, λ = 0.71073 Å
Hall symbol: -P 2ac 2ab	Cell parameters from 6089 reflections
*a* = 11.452 (2) Å	θ = 2.1–27.9°
*b* = 11.410 (2) Å	µ = 0.30 mm^−^^1^
*c* = 34.867 (7) Å	*T* = 113 K
*V* = 4555.8 (16) Å^3^	Block, colorless
*Z* = 16	0.22 × 0.20 × 0.16 mm
*F*(000) = 1800	

### Data collection

**Table:**

Rigaku Saturn diffractometer	4022 independent reflections
Radiation source: rotating anode	3619 reflections with *I* > 2σ(*I*)
confocal	*R*_int_ = 0.041
ω scans	θ_max_ = 25.0°, θ_min_ = 2.1°
Absorption correction: multi-scan (*CrystalClear*; Rigaku, 2000)	*h* = −13→13
*T*_min_ = 0.937, *T*_max_ = 0.953	*k* = −13→11
30046 measured reflections	*l* = −41→39

### Refinement

**Table:**

Refinement on *F*^2^	Primary atom site location: structure-invariant direct methods
Least-squares matrix: full	Secondary atom site location: difference Fourier map
*R*[*F*^2^ > 2σ(*F*^2^)] = 0.042	Hydrogen site location: inferred from neighbouring sites
*wR*(*F*^2^) = 0.119	H atoms treated by a mixture of independent and constrained refinement
*S* = 1.08	*w* = 1/[σ^2^(*F*_o_^2^) + (0.0667*P*)^2^ + 2.0145*P*] where *P* = (*F*_o_^2^ + 2*F*_c_^2^)/3
4022 reflections	(Δ/σ)_max_ = 0.001
268 parameters	Δρ_max_ = 0.30 e Å^−^^3^
3 restraints	Δρ_min_ = −0.28 e Å^−^^3^

### Special details

**Table:**

Geometry. All e.s.d.'s (except the e.s.d. in the dihedral angle between two l.s. planes) are estimated using the full covariance matrix. The cell e.s.d.'s are taken into account individually in the estimation of e.s.d.'s in distances, angles and torsion angles; correlations between e.s.d.'s in cell parameters are only used when they are defined by crystal symmetry. An approximate (isotropic) treatment of cell e.s.d.'s is used for estimating e.s.d.'s involving l.s. planes.
Refinement. Refinement of *F*^2^ against ALL reflections. The weighted *R*-factor *wR* and goodness of fit *S* are based on *F*^2^, conventional *R*-factors *R* are based on *F*, with *F* set to zero for negative *F*^2^. The threshold expression of *F*^2^ > σ(*F*^2^) is used only for calculating *R*-factors(gt) *etc*. and is not relevant to the choice of reflections for refinement. *R*-factors based on *F*^2^ are statistically about twice as large as those based on *F*, and *R*- factors based on ALL data will be even larger.

### Fractional atomic coordinates and isotropic or equivalent isotropic displacement parameters (Å^2^)

**Table:**

	*x*	*y*	*z*	*U*_iso_*/*U*_eq_	Occ. (<1)
Cl1	0.47805 (4)	1.03154 (4)	0.348689 (12)	0.02641 (15)	
Cl2	0.55772 (5)	0.75655 (5)	0.519211 (14)	0.03879 (17)	
N1	0.76243 (14)	0.70800 (14)	0.42512 (4)	0.0275 (4)	
N2	0.77675 (14)	0.78892 (14)	0.36959 (4)	0.0279 (4)	
N3	0.30575 (13)	0.63833 (13)	0.43738 (4)	0.0242 (3)	
N4	0.27524 (13)	0.73929 (13)	0.38612 (4)	0.0230 (3)	
C1	0.7808 (2)	0.6275 (2)	0.45759 (6)	0.0454 (6)	
H1A	0.7798	0.5481	0.4485	0.068*	
H1B	0.7196	0.6382	0.4761	0.068*	
H1C	0.8549	0.6437	0.4693	0.068*	
C2	0.81984 (17)	0.70586 (17)	0.39202 (5)	0.0290 (4)	
H2	0.8801	0.6547	0.3857	0.035*	
C3	0.68949 (18)	0.84657 (18)	0.38902 (6)	0.0315 (5)	
H3	0.6446	0.9085	0.3799	0.038*	
C4	0.68136 (17)	0.79632 (18)	0.42386 (6)	0.0312 (5)	
H4	0.6303	0.8175	0.4434	0.037*	
C5	0.8163 (2)	0.8133 (2)	0.33005 (6)	0.0432 (6)	
H5A	0.8946	0.7825	0.3267	0.052*	
H5B	0.8197	0.8975	0.3263	0.052*	
C6	0.73773 (18)	0.76060 (18)	0.30011 (5)	0.0284 (4)	
C7	0.76298 (19)	0.6509 (2)	0.28553 (6)	0.0393 (5)	
H7	0.8274	0.6097	0.2946	0.047*	
C8	0.6929 (2)	0.6021 (2)	0.25757 (7)	0.0520 (7)	
H8	0.7101	0.5280	0.2480	0.062*	
C9	0.5978 (2)	0.6625 (2)	0.24371 (6)	0.0494 (7)	
H9	0.5519	0.6302	0.2244	0.059*	
C10	0.5710 (2)	0.7711 (2)	0.25864 (7)	0.0474 (6)	
H10	0.5061	0.8116	0.2496	0.057*	
C11	0.6401 (2)	0.82016 (19)	0.28689 (6)	0.0374 (5)	
H11	0.6211	0.8931	0.2970	0.045*	
C12	0.2976 (2)	0.54811 (19)	0.46704 (6)	0.0358 (5)	
H12A	0.2647	0.5814	0.4899	0.054*	
H12B	0.3742	0.5182	0.4726	0.054*	
H12C	0.2487	0.4854	0.4582	0.054*	
C13	0.24416 (16)	0.64328 (16)	0.40510 (5)	0.0239 (4)	
H13	0.1888	0.5888	0.3972	0.029*	
C14	0.35863 (16)	0.79782 (16)	0.40711 (5)	0.0271 (4)	
H14	0.3952	0.8677	0.4004	0.032*	
C15	0.37723 (16)	0.73477 (17)	0.43921 (5)	0.0272 (4)	
H15	0.4288	0.7531	0.4589	0.033*	
C16	0.22392 (18)	0.77900 (18)	0.34989 (5)	0.0291 (4)	
H16A	0.1832	0.7141	0.3379	0.035*	
H16B	0.2859	0.8036	0.3327	0.035*	
C17	0.13971 (17)	0.87942 (16)	0.35559 (5)	0.0251 (4)	
C18	0.12909 (19)	0.96458 (18)	0.32750 (6)	0.0350 (5)	
H18	0.1755	0.9605	0.3057	0.042*	
C19	0.0500 (2)	1.05586 (19)	0.33155 (7)	0.0457 (6)	
H19	0.0432	1.1120	0.3123	0.055*	
C20	−0.0183 (2)	1.0634 (2)	0.36397 (8)	0.0463 (6)	
H20	−0.0710	1.1249	0.3668	0.056*	
C21	−0.00839 (19)	0.9798 (2)	0.39217 (7)	0.0389 (5)	
H21	−0.0541	0.9852	0.4141	0.047*	
C22	0.06960 (17)	0.88692 (17)	0.38806 (5)	0.0282 (4)	
H22	0.0748	0.8299	0.4070	0.034*	
O1	0.4899 (3)	0.9725 (3)	0.46644 (11)	0.0506 (9)	0.50
H1D	0.504 (5)	0.998 (4)	0.4438 (6)	0.061*	0.50
H1E	0.474 (5)	1.026 (3)	0.4830 (9)	0.061*	0.50

### Atomic displacement parameters (Å^2^)

**Table:**

	*U*^11^	*U*^22^	*U*^33^	*U*^12^	*U*^13^	*U*^23^
Cl1	0.0257 (3)	0.0269 (3)	0.0266 (2)	0.00190 (18)	0.00007 (17)	0.00296 (17)
Cl2	0.0373 (3)	0.0435 (3)	0.0356 (3)	0.0013 (2)	−0.0102 (2)	0.0026 (2)
N1	0.0301 (9)	0.0269 (9)	0.0255 (8)	−0.0025 (7)	−0.0035 (6)	0.0001 (6)
N2	0.0274 (9)	0.0304 (9)	0.0259 (8)	−0.0078 (7)	−0.0030 (6)	−0.0001 (7)
N3	0.0244 (8)	0.0234 (8)	0.0249 (8)	0.0034 (6)	0.0008 (6)	0.0020 (6)
N4	0.0228 (8)	0.0213 (8)	0.0249 (8)	0.0025 (6)	−0.0010 (6)	−0.0013 (6)
C1	0.0541 (15)	0.0445 (14)	0.0377 (11)	−0.0041 (11)	−0.0141 (10)	0.0141 (10)
C2	0.0242 (10)	0.0304 (11)	0.0325 (10)	0.0018 (8)	−0.0028 (8)	−0.0064 (8)
C3	0.0285 (10)	0.0271 (10)	0.0387 (11)	0.0012 (8)	−0.0084 (8)	−0.0028 (8)
C4	0.0241 (10)	0.0355 (11)	0.0339 (10)	0.0015 (8)	0.0009 (8)	−0.0080 (9)
C5	0.0435 (13)	0.0593 (15)	0.0270 (10)	−0.0251 (12)	0.0001 (9)	0.0043 (10)
C6	0.0309 (11)	0.0318 (10)	0.0225 (9)	−0.0094 (8)	0.0019 (8)	0.0029 (8)
C7	0.0292 (11)	0.0429 (13)	0.0457 (12)	0.0009 (10)	0.0079 (9)	−0.0036 (10)
C8	0.0530 (15)	0.0537 (15)	0.0493 (14)	−0.0125 (13)	0.0188 (12)	−0.0275 (12)
C9	0.0505 (15)	0.0755 (18)	0.0221 (10)	−0.0270 (14)	0.0028 (10)	−0.0111 (11)
C10	0.0398 (13)	0.0664 (17)	0.0360 (12)	−0.0086 (12)	−0.0101 (10)	0.0171 (12)
C11	0.0461 (13)	0.0303 (11)	0.0359 (11)	−0.0011 (10)	0.0002 (10)	0.0042 (9)
C12	0.0386 (12)	0.0356 (12)	0.0330 (10)	0.0047 (9)	0.0031 (9)	0.0123 (9)
C13	0.0224 (9)	0.0213 (9)	0.0279 (9)	−0.0004 (7)	0.0001 (7)	−0.0006 (7)
C14	0.0227 (9)	0.0226 (9)	0.0359 (10)	−0.0027 (8)	−0.0021 (8)	−0.0016 (8)
C15	0.0231 (10)	0.0285 (10)	0.0301 (10)	−0.0001 (8)	−0.0035 (8)	−0.0041 (8)
C16	0.0346 (11)	0.0323 (11)	0.0204 (9)	0.0038 (9)	0.0000 (8)	0.0003 (8)
C17	0.0264 (10)	0.0223 (9)	0.0267 (9)	−0.0013 (8)	−0.0067 (7)	0.0000 (7)
C18	0.0414 (12)	0.0306 (11)	0.0329 (10)	−0.0063 (9)	−0.0096 (9)	0.0080 (8)
C19	0.0552 (15)	0.0241 (11)	0.0580 (15)	−0.0024 (10)	−0.0319 (12)	0.0087 (10)
C20	0.0380 (13)	0.0302 (12)	0.0708 (17)	0.0085 (10)	−0.0283 (12)	−0.0149 (12)
C21	0.0266 (11)	0.0421 (13)	0.0479 (13)	0.0042 (9)	−0.0087 (9)	−0.0178 (11)
C22	0.0274 (10)	0.0285 (10)	0.0287 (9)	−0.0012 (8)	−0.0045 (8)	−0.0019 (8)
O1	0.055 (2)	0.041 (2)	0.056 (2)	−0.0050 (17)	0.0031 (18)	−0.0113 (16)

### Geometric parameters (Å, °)

**Table:**

N1—C2	1.328 (2)	C9—C10	1.379 (4)
N1—C4	1.371 (3)	C9—H9	0.9300
N1—C1	1.473 (2)	C10—C11	1.382 (3)
N2—C2	1.324 (3)	C10—H10	0.9300
N2—C3	1.375 (3)	C11—H11	0.9300
N2—C5	1.478 (2)	C12—H12A	0.9600
N3—C13	1.329 (2)	C12—H12B	0.9600
N3—C15	1.373 (2)	C12—H12C	0.9600
N3—C12	1.462 (2)	C13—H13	0.9300
N4—C13	1.328 (2)	C14—C15	1.348 (3)
N4—C14	1.376 (2)	C14—H14	0.9300
N4—C16	1.465 (2)	C15—H15	0.9300
C1—H1A	0.9600	C16—C17	1.511 (3)
C1—H1B	0.9600	C16—H16A	0.9700
C1—H1C	0.9600	C16—H16B	0.9700
C2—H2	0.9300	C17—C18	1.385 (3)
C3—C4	1.347 (3)	C17—C22	1.391 (3)
C3—H3	0.9300	C18—C19	1.387 (3)
C4—H4	0.9300	C18—H18	0.9300
C5—C6	1.504 (3)	C19—C20	1.378 (4)
C5—H5A	0.9700	C19—H19	0.9300
C5—H5B	0.9700	C20—C21	1.375 (4)
C6—C7	1.382 (3)	C20—H20	0.9300
C6—C11	1.387 (3)	C21—C22	1.393 (3)
C7—C8	1.380 (3)	C21—H21	0.9300
C7—H7	0.9300	C22—H22	0.9300
C8—C9	1.376 (4)	O1—H1D	0.860 (10)
C8—H8	0.9300	O1—H1E	0.857 (10)
			
C2—N1—C4	108.72 (16)	C9—C10—H10	119.8
C2—N1—C1	125.85 (18)	C11—C10—H10	119.8
C4—N1—C1	125.42 (18)	C10—C11—C6	120.0 (2)
C2—N2—C3	108.79 (16)	C10—C11—H11	120.0
C2—N2—C5	124.87 (19)	C6—C11—H11	120.0
C3—N2—C5	126.34 (18)	N3—C12—H12A	109.5
C13—N3—C15	108.77 (15)	N3—C12—H12B	109.5
C13—N3—C12	126.51 (17)	H12A—C12—H12B	109.5
C15—N3—C12	124.70 (16)	N3—C12—H12C	109.5
C13—N4—C14	108.73 (15)	H12A—C12—H12C	109.5
C13—N4—C16	125.25 (16)	H12B—C12—H12C	109.5
C14—N4—C16	125.93 (16)	N4—C13—N3	108.34 (16)
N1—C1—H1A	109.5	N4—C13—H13	125.8
N1—C1—H1B	109.5	N3—C13—H13	125.8
H1A—C1—H1B	109.5	C15—C14—N4	107.00 (17)
N1—C1—H1C	109.5	C15—C14—H14	126.5
H1A—C1—H1C	109.5	N4—C14—H14	126.5
H1B—C1—H1C	109.5	C14—C15—N3	107.15 (16)
N2—C2—N1	108.40 (17)	C14—C15—H15	126.4
N2—C2—H2	125.8	N3—C15—H15	126.4
N1—C2—H2	125.8	N4—C16—C17	112.14 (15)
C4—C3—N2	106.93 (18)	N4—C16—H16A	109.2
C4—C3—H3	126.5	C17—C16—H16A	109.2
N2—C3—H3	126.5	N4—C16—H16B	109.2
C3—C4—N1	107.16 (17)	C17—C16—H16B	109.2
C3—C4—H4	126.4	H16A—C16—H16B	107.9
N1—C4—H4	126.4	C18—C17—C22	118.81 (19)
N2—C5—C6	112.86 (17)	C18—C17—C16	119.67 (18)
N2—C5—H5A	109.0	C22—C17—C16	121.50 (17)
C6—C5—H5A	109.0	C17—C18—C19	120.8 (2)
N2—C5—H5B	109.0	C17—C18—H18	119.6
C6—C5—H5B	109.0	C19—C18—H18	119.6
H5A—C5—H5B	107.8	C20—C19—C18	120.1 (2)
C7—C6—C11	119.36 (19)	C20—C19—H19	120.0
C7—C6—C5	119.5 (2)	C18—C19—H19	120.0
C11—C6—C5	121.1 (2)	C21—C20—C19	119.8 (2)
C8—C7—C6	120.2 (2)	C21—C20—H20	120.1
C8—C7—H7	119.9	C19—C20—H20	120.1
C6—C7—H7	119.9	C20—C21—C22	120.5 (2)
C9—C8—C7	120.5 (2)	C20—C21—H21	119.8
C9—C8—H8	119.8	C22—C21—H21	119.8
C7—C8—H8	119.8	C17—C22—C21	120.04 (19)
C8—C9—C10	119.5 (2)	C17—C22—H22	120.0
C8—C9—H9	120.2	C21—C22—H22	120.0
C10—C9—H9	120.2	H1D—O1—H1E	114.7 (19)
C9—C10—C11	120.4 (2)		
			
C3—N2—C2—N1	0.4 (2)	C14—N4—C13—N3	−0.5 (2)
C5—N2—C2—N1	−178.98 (17)	C16—N4—C13—N3	−177.32 (16)
C4—N1—C2—N2	−0.8 (2)	C15—N3—C13—N4	0.7 (2)
C1—N1—C2—N2	177.98 (18)	C12—N3—C13—N4	178.88 (17)
C2—N2—C3—C4	0.2 (2)	C13—N4—C14—C15	0.1 (2)
C5—N2—C3—C4	179.59 (18)	C16—N4—C14—C15	176.88 (16)
N2—C3—C4—N1	−0.7 (2)	N4—C14—C15—N3	0.4 (2)
C2—N1—C4—C3	1.0 (2)	C13—N3—C15—C14	−0.7 (2)
C1—N1—C4—C3	−177.85 (18)	C12—N3—C15—C14	−178.89 (17)
C2—N2—C5—C6	99.5 (2)	C13—N4—C16—C17	103.1 (2)
C3—N2—C5—C6	−79.8 (3)	C14—N4—C16—C17	−73.2 (2)
N2—C5—C6—C7	−93.2 (2)	N4—C16—C17—C18	147.06 (18)
N2—C5—C6—C11	86.7 (3)	N4—C16—C17—C22	−34.8 (3)
C11—C6—C7—C8	1.2 (3)	C22—C17—C18—C19	0.0 (3)
C5—C6—C7—C8	−178.9 (2)	C16—C17—C18—C19	178.16 (19)
C6—C7—C8—C9	0.5 (3)	C17—C18—C19—C20	0.7 (3)
C7—C8—C9—C10	−1.6 (4)	C18—C19—C20—C21	−0.4 (3)
C8—C9—C10—C11	1.1 (3)	C19—C20—C21—C22	−0.5 (3)
C9—C10—C11—C6	0.7 (3)	C18—C17—C22—C21	−0.9 (3)
C7—C6—C11—C10	−1.8 (3)	C16—C17—C22—C21	−179.04 (18)
C5—C6—C11—C10	178.28 (19)	C20—C21—C22—C17	1.2 (3)

### Hydrogen-bond geometry (Å, °)

**Table:**

*D*—H···*A*	*D*—H	H···*A*	*D*···*A*	*D*—H···*A*
O1—H1E···O1^i^	0.86 (1)	1.81 (4)	2.434 (7)	128 (4)
O1—H1E···Cl2^i^	0.86 (1)	2.51 (3)	3.179 (3)	135 (4)

Symmetry codes: (i) −*x*+1, −*y*+2, −*z*+1.
